# Metabolic Optimisation in Total Joint Arthroplasty: A Single-Centre Retrospective Cohort Pilot Study on the Safety and Feasibility of a Digitally Supported Perioperative Diet Modification

**DOI:** 10.3390/jcm15051948

**Published:** 2026-03-04

**Authors:** Hwee Wen Ong, Khairul Anwar Ayob, David Siew-Kit Choon, Virginia Hartono

**Affiliations:** 1 National Orthopaedic Centre of Excellence for Research and Learning, Department of Orthopaedic Surgery, Faculty of Medicine, University of Malaya, Kuala Lumpur 50603, Malaysia; 2 Medical Education Research Development Unit, University of Malaya, Kuala Lumpur 50603, Malaysia; 3 Quill Orthopaedic Specialist Centre, Kuala Lumpur 60000, Malaysia

**Keywords:** diabetes, digital health, low-carbohydrate diet, perioperative optimisation, total joint arthroplasty, weight loss

## Abstract

**Background/Objectives**: Obesity and type 2 diabetes are increasingly common among patients undergoing hip and knee arthroplasty and are associated with higher risks of prosthetic joint infection, impaired wound healing, and prolonged hospitalisation. Dietary carbohydrate restriction has demonstrated benefits in glycemic control and weight reduction, but its feasibility and safety in the perioperative arthroplasty population remain underexplored. This pilot study evaluated the safety, feasibility, and short-term metabolic effects of a low-carbohydrate diet supported by WhatsApp-based meal photo-logging in patients undergoing total hip or knee arthroplasty. **Methods**: A retrospective cohort analysis was performed on 43 patients enrolled in a carbohydrate-restricted dietary programme between 2021 and 2024. Patients submitted photographs of all meals via WhatsApp with a minimum contact frequency of four times daily, enabling real-time feedback and medication adjustment. Anthropometric and metabolic parameters, including weight, BMI, HbA1c, renal function, and lipid profile, were assessed before and after the intervention. **Results**: Participants (mean age 69.12 ± 7.51 years) demonstrated significant improvement across several metabolic markers. Mean weight decreased by 5.74 kg (*p* < 0.001), BMI by 2.26 kg/m^2^ (*p* < 0.001), and HbA1c by 0.72% (*p* < 0.001). No episodes of severe hypoglycaemia or perioperative discharge delays related to glycemic instability were observed. Renal function remained stable, with no significant change in eGFR (*p* = 0.442). Among patients with available lipid data (*n* = 14), LDL-cholesterol and total cholesterol increased, while triglycerides showed a non-significant downward trend. **Conclusions**: A low-carbohydrate diet combined with high-frequency digital monitoring appears feasible and safe, achieving meaningful short-term improvements in weight and glycemic control without adverse renal or hypoglycemic events. The lipid changes observed, however, warrant cautious interpretation. These findings are hypothesis-generating, and larger prospective studies are needed to confirm the clinical impact of this approach and its relevance to perioperative optimisation.

## 1. Introduction

Diabetes represents a major global health challenge, with the IDF Diabetes Atlas (2024) reporting that approximately 1 in 9 adults aged 20–79 years currently lives with diabetes worldwide and projecting a substantial further increase by 2050, with four in five affected individuals residing in low- and middle-income countries [1]. In Malaysia, this burden is particularly pronounced: national data indicate an age-standardised adult diabetes prevalence of about 21%, alongside a high and rising prevalence of obesity documented in the Malaysian Cohort and reflected in the Ministry of Health’s Clinical Practice Guidelines on obesity management [1,2].

These metabolic comorbidities are highly relevant to hip and knee arthroplasty, as obesity and suboptimal glycaemic control have been associated with increased risks of prosthetic joint infection, impaired wound healing, cardiovascular events, and prolonged length of stay. Several large arthroplasty studies have demonstrated that elevated preoperative HbA1c and perioperative hyperglycaemia correlate with higher rates of postoperative infection and early complications [3,4,5,6,7,8,9].

In response, orthopaedic and endocrine guidelines—such as the AAOS Clinical Practice Guideline on the management of knee osteoarthritis and recommendations on perioperative diabetes care—now emphasise optimisation of weight and glycaemic control before elective arthroplasty, often advocating weight loss and postponement of surgery when HbA1c exceeds defined thresholds [2,3,4,6,7,8,9].

However, achieving stable glucose levels in the perioperative period remains challenging because of stress-induced insulin resistance, variability in perioperative carbohydrate intake, and the limitations of traditional sliding-scale insulin protocols [8,10,11]. These challenges have prompted growing interest in low-carbohydrate and ketogenic dietary strategies, which have demonstrated improvements in weight and HbA1c in patients with type 2 diabetes, and in digital health tools such as smartphone-based coaching, mobile social networking applications, and Continuous Glucose Monitoring as potential adjuncts for safer and more effective perioperative metabolic optimisation in total joint arthroplasty candidates [5,12,13,14,15,16,17,18,19,20,21].

## 2. Materials and Methods

### 2.1. Study Design and Population

**Figure 1:**
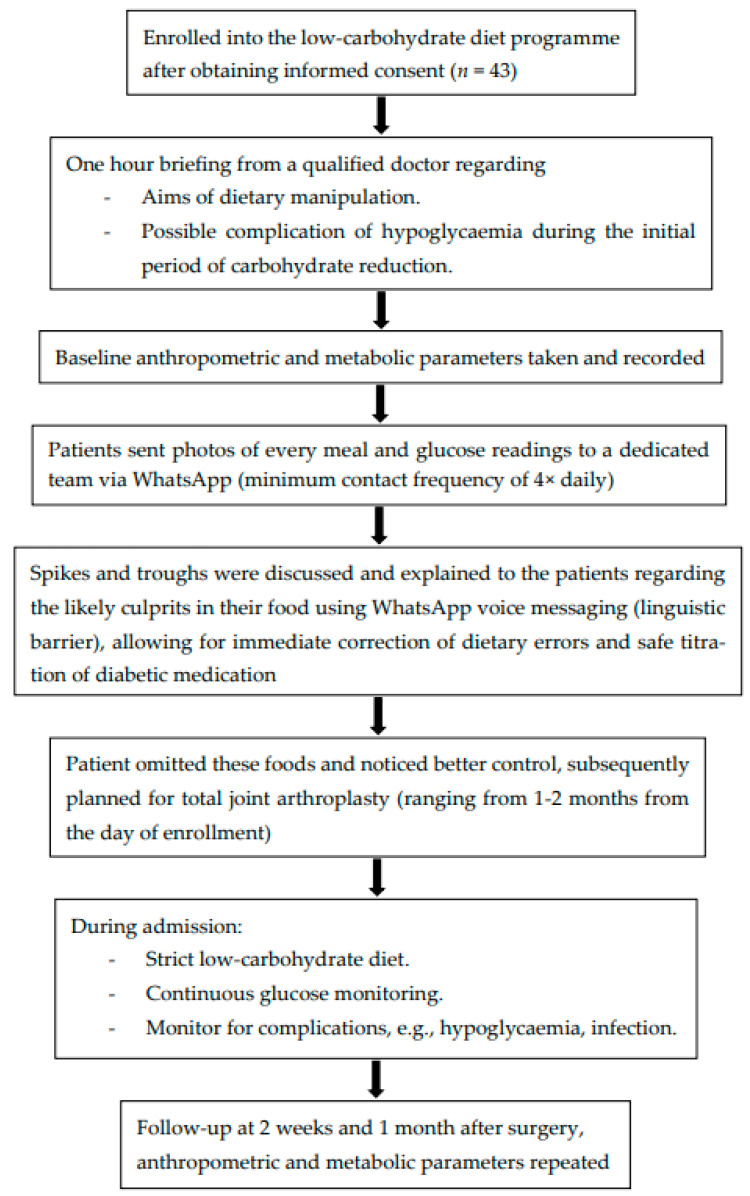
Flow diagram depicting clinical protocol and perioperative intervention pathway.

This study follows a retrospective cohort design analysing data from patients prospectively enrolled in a clinical quality improvement programme at a single centre between 2021 and 2024. The study was conducted in accordance with current legislation and ethical standards for clinical research and was approved by the Medical Research Ethics Committee of University of Malaya Medical Centre on 22 June 2025 (MREC ID NO.: 2025320-14882). Patients were recruited into the low-carbohydrate diet programme in Arthro Associates Clinic and underwent elective primary TJA (hip or knee) in Quill Orthopaedic Specialist Centre (Figure 1). Informed consent was obtained from all participants.

Inclusion criteria were those aged ≥ 18 years; type II diabetes or overweight patients; patients who underwent total joint arthroplasty (hip or knee); and those who were willing and able to provide informed consent. The exclusion criteria were those aged < 18 years; type I diabetes; history of bariatric surgery for weight loss; haemoglobin < 11 mg/dL; recent blood donation or blood transfusion (self-reported, past 4 months); human immunodeficiency virus (self-report); self-reported history of intensive care unit stays due to COVID-19 3 months prior to initiation of dietary plan; and, for women, pregnancy and breastfeeding [12].

Rationale for Exclusions: Anaemia, recent blood loss, and HIV were excluded, as these factors may lead to falsely altered HbA1c readings. A severe, recent COVID-19 diagnosis was excluded, as it may independently dysregulate blood glucose levels.

### 2.2. Intervention Protocol

1. Dietary Substrate: The dietary intervention followed a low-carbohydrate “plate method” framework rather than strict calorie counting. Patients were instructed to consume the following: one portion of carbohydrate, one portion of protein ~40 g (fish or chicken), and one portion of simple protein (egg or tofu) and vegetables, with a calorie count between 300 and 400 kcal. Rice was replaced with cauliflower rice.
2. Digital Liaison: Patients were required to send photos of every meal and glucose readings to a dedicated team via WhatsApp when they were in the programme, lasting between 2 and 4 months.
3. Frequency: A minimum contact frequency of 4× daily was enforced. Dietary adherence was assessed qualitatively by the clinical team based on the visual composition of meal photos. Immediate correction of dietary errors involved the use of WhatsApp voice messaging to overcome potential linguistic barriers. Clinicians would identify specific glycemic culprits in the photos that correlated with observed glucose spikes, allowing for real-time behavioural modification.
4. Monitoring: A transition from finger-prick testing to Continuous Glucose Monitoring (CGM) was implemented preoperatively to capture glycemic variability. Diabetic medication adjustments were performed based on the clinical team’s discretion, guided by daily glucose trends.

### 2.3. Primary and Secondary Outcomes

1. Primary: (a) Occurrence of severe hypoglycaemia, and (b) discharge delay due to glycaemic instability.
2. Secondary: Changes in weight, BMI, HbA1c, FBS, lipid and renal parameters, and perioperative RBS range.

### 2.4. Statistical Analysis

Data was collected and managed using Microsoft Excel and analysed using IBM SPSS Statistics (version 31) software. Descriptive statistics were utilised to summarise the data. Continuous variables were expressed as mean ± standard deviation or median with interquartile range (IQR). Categorical variables were presented as frequencies and percentages. Differences in baseline characteristics were analysed using independent and paired sample *t*-tests, while categorical data were compared using Fisher's exact test. A *p*-value of <0.05 was taken as statistically significant.

## 3. Results

### 3.1. Patient Demographic

The mean age of our study population is 69.12 ± 7.5 years, with 69.8% of them female. A total of 23 patients underwent bilateral TKR, 14 underwent unilateral TKR and 6 underwent unilateral THR. A total of 28 patients were diabetic, of whom 24 were on oral agents, and 4 were on insulin. The rest were under the non-diabetic/overweight category. Two patients defaulted from the full protocol but were included in the intention-to-treat analysis, where data permitted (Table 1).

Many patients were able to reduce or stop diabetic medications after the low-carbohydrate intervention. Among the 26 patients on OHA, 11 patients (42.3%) reduced the number of OHA used, and 7 patients (26.9%) were able to completely stop all OHA. This indicates that about 69% either reduced or discontinued their oral diabetic medications, consistent with improved glycaemic control.

Among the four patients on insulin, one patient reduced the insulin dose, and three patients were able to stop insulin completely and switch to OHA (Table 2). This suggests that, in this small insulin-treated subgroup, most patients improved enough to de-escalate from insulin to oral therapy, which supports the metabolic effectiveness of the intervention while maintaining safety.

### 3.2. Anthropometric and Metabolic Efficacy

The intervention delivered rapid, statistically significant optimisation (Table 3):

- Weight: Mean loss of 5.74 ± 4.10 kg (*p* < 0.001), ranging from 0.7 to 22.0 kg.
- BMI: Reduction of 2.26 ± 1.47 kg/m^2^ (*p* < 0.001), ranging from 0.27 to 7.18 kg/m^2^.
- HbA1c: Improved by 0.72 ± 0.49% (*p* < 0.001), ranging from −0.2–1.9%, with the highest HbA1c post-intervention recorded at 7.2%

**Table 3:** Parameters measured pre- and post-intervention.

Parameters	Before	After	Difference	*p*-Value	Total (n)
Height (m)		1.58 ± 0.08		-	43
Weight (kg)	75.13 ± 17.18	69.38 ± 15.21	5.74 ± 4.10	<0.001	43
BMI (kg/m^2^)	29.86 ± 5.66	27.60 ± 5.16	2.26 ± 1.47	<0.001	43
Waist circumference	38.54 ± 6.05	34.71 ± 4.65	3.83 ± 2.14	<0.001	18
HbA1c	6.54 ± 0.70	5.82 ± 0.49	0.72 ± 0.49	<0.001	24
FBS	7.21 ± 1.88	5.91 ± 0.84	1.30 ± 2.12	0.002	27
Perioperative RBS	7.12 ± 2.20	10.08 ± 2.50	2.96 ± 2.88	<0.001	36
Total cholesterol	4.74 ± 1.02	5.45 ± 1.46	−0.71 ± 1.24	0.026	14
Triglyceride	1.88 ± 0.86	1.50 ± 0.65	0.38 ± 0.89	0.065	14
HDL	1.49 ± 0.43	1.37 ± 0.33	0.12 ± 0.22	0.032	14
LDL	2.79 ± 0.87	3.60 ± 1.37	−0.81 ± 1.20	0.012	14
Urea	7.41 ± 3.44	9.01 ± 4.51	−1.59 ± 3.36	0.030	18
Creatinine	101.09 ± 33.25	92.65 ± 26.26	8.44 ± 25.76	0.080	20
eGFR	62.31 ± 23.94	62.94 ± 20.40	−0.63 ± 16.91	0.442	16

Baseline anthropometric and metabolic parameters were taken and recorded during enrollment. During follow-up after surgery, ranging from 1 to 3 months, these parameters were repeated.

### 3.3. The Safety Profile

Safety was the primary endpoint of this pilot study.

- Hypoglycaemia: Despite 27 patients being on diabetic medication, none experienced dangerous hypoglycaemia (<4.0 mmol/L) requiring rescue intervention.
- Discharge Delays: None of the patients had their discharge delayed due to glycaemic instability.
- Renal Stability: While Urea increased significantly (*p* = 0.030), likely due to protein turnover, Creatinine (*p* = 0.080) and eGFR (*p* = 0.442) showed no significant change. This confirms that renal filtration function was preserved.

### 3.4. The Lipid Profile

The data revealed a distinct divergence in lipid markers:

- LDL-C: Increased significantly by 0.81 mmol/L (*p* = 0.012).
- Total Cholesterol: Increased significantly (*p* = 0.026).
- Triglycerides: Remained low (1.50 ± 0.65 mmol/L) with a trend toward reduction (*p* = 0.065).
- HDL: Decreased modestly but remained within generally favourable ranges (1.37 mmol/L).

The most critical finding for the orthopaedic community is the absence of life-threatening hypoglycaemia. In standard care, the combination of carbohydrate intake (raising sugar) and insulin (lowering sugar) creates glycaemic volatility. This approach may have contributed to a more stable perioperative glycaemia, preventing perioperative discharge delays and rendering it cost-effective.

## 4. Discussion

To our knowledge, this retrospective pilot study is among the first to examine the use of a carbohydrate-restricted dietary intervention supported by high-frequency WhatsApp monitoring for patients with type 2 diabetes or obesity undergoing hip and knee arthroplasty. The findings suggest that this combined approach is feasible and may contribute to improvements in weight, glycaemic control and several perioperative metabolic parameters.

Metabolic comorbidities such as obesity and diabetes are well-established risk factors for perioperative complications, notably prosthetic joint infection (PJI), the most devastating complication in arthroplasty. Furthermore, glycemic variability—the rapid fluctuation between high and low blood sugar—has been identified as a potent pro-inflammatory stressor that impairs wound healing, increasing length of hospital stay.

Traditional perioperative glycaemic management relied on reactive insulin titration. Patients are often admitted with suboptimal metabolic parameters, and hyperglycaemia is managed perioperatively using sliding-scale insulin. This approach presents a dual risk:

1. Inefficacy: It treats the symptom (high sugar) without addressing the root cause (insulin resistance/dietary input).
2. Safety: Aggressive insulin therapy increases the risk of iatrogenic hypoglycaemia, which is associated with arrhythmias, falls, and increased mortality in the elderly.

In contrast, dietary carbohydrate restriction targets the primary driver of hyperglycaemia and may offer a more physiologic method to mitigate perioperative glycaemic volatility. In this study, patients achieved significant reductions in body weight and HbA1c over a short preoperative window without observed episodes of severe hypoglycaemia, supporting the potential safety of this approach.

A notable component of this intervention was the use of a WhatsApp-based “tight feedback loop,” requiring patients to submit meal photographs and glucose readings several times daily. This strategy appeared to enhance engagement and adherence, demonstrating that patients can successfully utilise simple digital tools when appropriately guided [15,16,17,18,19]. Digital health applications have been increasingly recognised as valuable adjuncts in chronic disease management, and the present study extends this concept into the preoperative optimisation phase for major orthopaedic surgery.

This pilot study aimed to:

1. Establish Safety: Verify that strict carbohydrate restriction does not cause hypoglycaemia or renal impairment in a surgical cohort.
2. Evaluate Feasibility: Assess the efficacy of WhatsApp-based photo-logging for patients.
3. Analyse Lipid Physiology: Interpret the “Lipid Paradox” (rising LDL with weight loss) through the lens of the Lipid Energy Model [22].

Major orthopaedic surgery induces a hypermetabolic stress response characterised by the release of cortisol, catecholamines, and glucagon. This state induces transient insulin resistance, often termed “stress hyperglycaemia.” This explains the increase in perioperative random blood sugar post-surgery. In patients with poor baseline metabolic health, this response is exaggerated, significantly increasing the risk of deep infection. Despite this, this intervention shows glycemic resilience during the immediate recovery phase, where there were minor fluctuations in perioperative blood sugar.

We observed a “Lipid Paradox” characterised by elevated Low-Density Lipoprotein Cholesterol (LDL-C) concomitant with weight loss and improved glycemic markers, as the ketogenic diet shifts the body’s primary fuel source from glucose to ketones and fatty acids. This phenomenon has been described in other carbohydrate-restriction studies. Theoretical frameworks such as the Lipid Energy Model [22] propose that in a carbohydrate-restricted state, the liver packages triglycerides into very-low-density lipoproteins (VLDLs) to fuel peripheral tissues. As lean tissue (muscle) rapidly hydrolyses these triglycerides for energy, the VLDL particle shrinks and becomes an LDL particle.

In this context, elevated LDL is a “remnant” of high-energy trafficking (fat burning), not a marker of broken cholesterol metabolism. The profile of High LDL + Low Triglycerides + High HDL is characteristic of the Lean Mass Hyper-Responder phenotype, which differs fundamentally from the atherogenic triad (High TG, Low HDL, Small LDL) associated with cardiovascular disease. HDL levels in our cohort decreased slightly, which diverges from typical responses to weight loss. A 2024 expert review summarising multiple trials reports that during active weight reduction, HDL often shows no change or a decrease, while after weight stabilises, HDL returns to baseline or increases [23]. Given the small sample size for lipid data, these findings should be viewed as hypothesis-generating.

The success of this intervention relied on “Digital Dosage.” The requirement to photograph every meal created a Hawthorne Effect (behaviour change due to observation). This transformed the diet from a static prescription into a dynamic, managed process, suggesting that patients can successfully engage with digital health tools [15,16,17,18,19].

This study also has several limitations inherent to its retrospective design. Firstly, the lack of a control group receiving standard care limits causal inference. Selection bias is also possible, as individuals who agreed to intensive dietary monitoring may represent a highly motivated subset of patients with higher baseline digital literacy than the general arthroplasty population. Missing data, particularly for lipid and renal parameters, reduces the robustness of some conclusions. Finally, the dietary intervention was not strictly standardised, and meal assessments relied on photographs rather than objective nutritional analysis.

Despite these limitations, the results support the feasibility of implementing a digitally supported low-carbohydrate diet in the perioperative setting and suggest potential metabolic benefits that may justify further study. Prospective, controlled trials with larger sample sizes, complete biochemical data, and longer postoperative follow-up will be necessary to determine the true clinical impact of this protocol on surgical outcomes, complication rates, and healthcare utilisation.

## 5. Conclusions

A low-carbohydrate diet combined with high-frequency digital monitoring appears feasible and safe, achieving meaningful short-term improvements in weight and glycemic control without adverse renal or hypoglycemic events. The lipid changes observed warrant cautious interpretation. These findings are hypothesis-generating, and larger prospective studies are needed to confirm the clinical impact of this approach and its relevance to perioperative optimisation.

## Figures and Tables

**Table 1:** Patient demographic.

Demographics			Total (*n* = 43)
Age	69.12 ± 7.51		43
Gender	Male	13 (30.2%)	43
	Female	30 (69.8%)	
Category	Non-diabetic	13 (30.2%)	43
	Diabetic (not on insulin)	24 (55.8%)	
	Diabetic (on insulin)	4 (9.3%)	
	Defaulter	2 (4.7%)	
Surgery	Unilateral TKR	14 (32.6%)	43
	Bilateral TKR	23 (53.5%)	
	Unilateral THR	6 (14.0%)	
Medication	Not on diabetic medication	13 (30.2%)	43
	OHA	26 (60.5%)	
	Insulin	4 (9.3%)	

**Table 2:** Changes in oral hypoglycaemic agents and insulin requirements following the low-carbohydrate intervention.

Medication			Total (*n*)	*p*-Value
OHA	No change in the number of OHA	8 (30.8%)	26	
	Reduction in the number of OHA	11 (42.3%)		<0.001
	Complete stop of all OHA	7 (26.9%)		
Insulin	Reduction in insulin units	1 (25%)	4	
	Complete stop of insulin, change to OHA	3 (75%)		0.006

## Data Availability

The original contributions presented in this study are included in the article. Further inquiries can be directed to the corresponding author.

## Abbreviations

The following abbreviations are used in this manuscript:

AAOS	American Academy of Orthopaedic Surgeons
BMI	Body mass index
CGM	Continuous Glucose Monitoring
CPG	Clinical Practice Guidelines
eGFR	Renal filtration
FBS	Fasting blood sugar
HbA1c	Glycated haemoglobin
HDL	High-density lipoprotein
IDF	International Diabetes Federation
IQR	Interquartile range
LDL	Low-density lipoprotein
LEM	Lipid Energy Model
OHA	Oral hypoglycaemic agents
RBS	Random blood sugar
TG	Triglyceride
THR	Total hip replacement
TJA	Total joint arthroplasty
TKR	Total knee replacement
TMC	The Malaysian Cohort
PJI	Prosthetic joint infection
RCT	Randomised controlled trial
VLDL	Very-low-density lipoprotein
